# Digital technology adoption in livestock production with a special focus on ruminant farming

**DOI:** 10.1017/S1751731120001391

**Published:** 2020-11

**Authors:** T. Groher, K. Heitkämper, C. Umstätter

**Affiliations:** Research Division on Competitiveness and System Evaluation, Agroscope, Tänikon 1, 8356 Ettenhausen, Switzerland

**Keywords:** precision livestock farming, ruminants, survey, small-scale farming, farm characteristics

## Abstract

Digitalisation is an integral part of modern agriculture. Several digital technologies are available for different animal species and form the basis for precision livestock farming. However, there is a lack of clarity as to which digital technologies are currently used in agricultural practice. Thus, this work aims to present for the first time the status quo in Swiss livestock farming as an example of a highly developed, small-scale and diverse structured agriculture. In this context, the article focuses on the adoption of electronic sensors and measuring devices, electronic controls and electronic data-processing options and the usage of robotics in ruminant farming, namely, for dairy cattle, dairy goats, suckler cows, beef cattle and meat-sheep. Furthermore, the use of electronic ear tags for pigs and the smartphone usage for barn monitoring on poultry farms was assessed. To better understand the adoption process, farm and farmer’s characteristics associated with the adoption of (1) implemented and (2) new digital technologies in ruminant farming were assessed using regression analyses, which is classified at a 10% adoption hurdle. The results showed clear differences in the adoption rates between different agricultural enterprises, with both types of digital technologies tending to be used the most in dairy farming. Easy-to-use sensors and measuring devices such as those integrated in the milking parlour were more widespread than data processing technologies such as those used for disease detection. The husbandry system further determined the use of digital technologies, with the result that farmers with tie stall barns were less likely to use digital technologies than farmers with loose housing systems. Additional studies of farmers’ determinants and prospects of implementation can help identify barriers in the adoption of digital technologies.

## Implications

This work shows the recent digital technology adoption in Swiss livestock farms with a special focus on ruminant farming. The use of digital technologies forms the basis for a successful, large-scale implementation of precision livestock farming in practice. Switzerland is used as an example for highly developed, small-scaled European agriculture. The results allow an international country-specific comparison of the spread of digital technologies in different livestock farming enterprises. In addition, typical farm and farmers’ characteristics associated with the adoption of different types of digital technologies could be identified.

## Introduction

Precision livestock farming (**PLF**) incorporates the use of digital technologies. By precisely controlling agricultural processes, it aims for improving production and reproduction, increasing human and animal welfare and facilitating targeted resource use to reduce the environmental impact. The basis for PLF is the use of digital technologies that record animal individual, animal group-related or environmental parameters (Wathes *et al.*, 2008; Banhazi *et al.*, 2012; Berckmans, 2017).

The application of technologies has made every day work easier in the agricultural sector. An example is the milking process, which, in brief, shifted from hand milking to machine milking during the last 150 years. The further development in this area has then led to the introduction of milking robots in the 1980s, which brought new advantages for farmers such as labour efficiency and the automatic recording of several animal-related parameters (Ordolff, 2001).

The increasing numbers of animals per farm and the awareness of animal- and environmental-friendly production systems with decreased resource use call for new solutions, which could be found in digital technologies used in the entire livestock farming system (Berckmans, 2006). However, the overall picture is that the adoption of digital technologies varies widely across the different technologies, animal species and areas of application. For example, digital technologies in the milking sector, such as sensors for measuring milk quantity or automatic concentrate feeders are widely used and have been available for decades (Ordolff, 2001). In addition, technologies in poultry production such as egg counting, bird weighing or environmental and feeding controls are commercially implemented (Banhazi *et al.*, 2012). In contrast, there are technologies that have been available for a long-time but are still not implemented on a large-scale into livestock farming. Examples are animal tracking systems, automatic heat detection or automatic milking systems, which we further refer to as milking robots (Borchers and Bewley, 2015; Edwards *et al.*, 2015; Gargiulo *et al.*, 2018).

We know from the literature that socio-demographic factors are correlated with technology adoption and that some farm types or enterprises are more likely than others to use modern technologies, although these results mainly originate from investigations on crop farms (Pierpaoli *et al.*, 2013). Potential farm and farmer’s characteristics related to technology adoption are farm size, the production system (organic or conventional), farm specialisation or the farmer’s age (Tey and Brindal, 2012; Pierpaoli *et al.*, 2013; Paustian and Theuvsen, 2017). The results are not always consistent and vary with regard to the type of technology (Konrad *et al.*, 2019) and the investigated country (Tamirat *et al.*, 2018; Barnes *et al.*, 2019). For example, whereas Lima *et al.* (2018) found age or farm size not being associated with the adoption of electronic identification tools by commercial sheep farmers, Konrad *et al.* (2019) found that adoption of nutrient abatement technologies increased with increasing farm sizes and decreased for older farmers. Barkema *et al.* (2015) summarised the adoption rates of milking robots in selected countries and showed strong differences worldwide. Whereas in Denmark and Sweden more than 20% of dairy farmers had adopted milking robots, the adoption rate was lower in other countries with between 15% and 20% in Iceland and the Netherlands, between 10% and 15% in Norway and less than 10% in Finland, Germany and Canada. However, the sampling procedure was very heterogeneous between the studies and mostly non-representative.

However, a crucial difference in the adoption of digital technologies in the livestock sector compared with plant production is that husbandry systems are less flexible and are planned ahead for decades. One reason for it could lie in the high investment costs and the longevity of investment. For example, the investment costs for a new dairy cattle barn in Switzerland amount to about 11 000 to 22 000 Swiss francs per cow place depending on, for example, the type of barn, the number of cows or the milking parlour type, with a payback period of 25 years (Gazzarin and Hilty, 2002). In addition, the type of husbandry can determine the use of digital technologies. An example is the use of activity sensors for individual animal monitoring: Whereas animal well-being can be derived from the data in loose housing systems, the implementation of this technology in tie stall barns is not adequate because here cows cannot express their behaviour freely.

Livestock production is a main part of Swiss agriculture because the topographical and climatic conditions are well suited for meadows and pastures. In 2016, 74% of farms were specialised in livestock production and the area for fodder crops comprised 70% of the total agricultural area (FSO, 2017). Compared with its neighbour countries such as Germany or France, the average farm size is small with about 20.5 ha in 2018 (Ferjani *et al.*, 2015; FOAG, 2018). The Swiss Federal Government financially supports sustainable agriculture, allowing small, diversified farms to be maintained. However, the worldwide trend toward larger specialised farms is also appearing in Switzerland, resulting in decreasing numbers of farms and increasing farm sizes. In 2018, a farmer managed on average more than twice as much area as in 1975, which increased the number of farms in the upper farm size distribution of 50 ha and more. Also the number of animals per farm increased (FSO, 2019a). However, there are legal maximum levels, which limit the number of animals per farm, according to the regulation on maximum stock in meat and egg production of the Swiss Federal Council. Common husbandry systems for dairy cattle in Switzerland are loose housing systems and tie stall barns, even though the proportion for the latter is declining (Schrade, 2009). Suckler cows, beef cattle, goats and sheep are usually kept in loose housing systems.

Our study focused on two questions: (1) Which digital technologies are currently used in Swiss livestock farming? (2) Which farm and farmers’ characteristics are associated with the adoption with a special focus on ruminant farming? In this context, digital technologies included all queried technologies such as electronic sensors and measuring devices (**ESMDs**), electronic controls (**ECs**) and electronic data-processing options (**EDPOs**) as well as robotics, electronic ear tags and smartphone usage. Here, Swiss livestock farms were investigated as an example of small-scale and diversely structured agriculture, where livestock farming is one of the most important enterprises. The article evaluates the status quo of technology adoption based on a representative, large-scale survey with randomly sampled farmers situated across Switzerland. In a first step, frequencies of digital technology adoption in ruminant, pig and poultry farming were evaluated. Based on the survey results, the digital technologies were classified into implemented ones that have already been proven in practice and new ones that make the farmers pioneers in their use. In a second step, the farm and farmers’ characteristics associated with the adoption of both technology types in ruminant farming were identified.

## Material and methods

### Data collection

This work was part of a comprehensive written postal survey among Swiss farmers with the aim to assess the current state of mechanisation and automation in Swiss agriculture for labour-economic evaluations. For this purpose, specific questionnaires for 17 different types of agricultural enterprises were developed to cover the typical machinery usage and working procedures accruing in each enterprise. The questionnaires contained different numbers of questions and answer options, which are relevant to Swiss agriculture. The farmers were asked to specify only work to be done on their own farm and the respective agricultural enterprise, even if they had more than one enterprise (e.g. if a farmer had dairy cattle and meat-sheep, the questionnaire was only related to one of the two enterprises).

The sampling plan was developed by the Federal Statistical Office’s Statistical Methods Section to draw a random sample from the overall farm population. Based on the Swiss Farm Structure Survey (**FSS**) from 2016, separate sampling populations were defined for the 17 agricultural enterprises. Therefore, a cut-off was determined based on the size in hectare or number of animals, and a stratification was created to ensure that all farm sizes were considered within the sample. The annually conducted FSS includes almost all Swiss farms and contains information on socio-demographic aspects and technical and structural factors (Ferjani *et al.*, 2015; FSO, 2016). Because there are multiple official languages in Switzerland, the questionnaires were available in German, French and Italian.

In total, 4954 written questionnaires (about 10% of all Swiss farms) were sent to farmers located across Switzerland during January to March 2018. Because our study focused on digital technology adoption on livestock farms, 1497 returned questionnaires from the following enterprises were considered in this article: dairy cattle, dairy goats, suckler cows, beef cattle, meat-sheep, breeding pigs, fattening pigs, laying hens and broilers.

In addition, for ruminants the frequencies of adoption of ESMDs, ECs with a central computer, EDPOs and robotics were assessed. Furthermore, the adoption of digital technologies for pigs and poultry was evaluated using two example technologies: electronic ear tags for pigs and smartphone usage for poultry barn monitoring. For most questions, multiple answers were possible. The answer options included various sensors and applications from the thematic areas of feeding, animal behaviour and activity, animal monitoring and identification and, if applicable, milking technologies.

### Farm and farmers’ characteristics

To better understand the adoption process, farm and farmers’ characteristics related to the adoption of digital technologies in Swiss ruminant farming were examined. Relevant farm variables from the FSS data were linked to the respective farms from the questionnaires. The following variables were considered for further analyses: the continuous variables ‘age’, ‘agricultural area’ and ‘number of livestock units’, the dichotomous variables ‘gender (male/female)’, ‘production system (conventional/organic)’ and ’on-farm working time (part-time/full-time)’ and the polytomous variables ‘zone’, ‘region’, ‘main farm type’ and ‘barn system’. The zones were divided into ‘valley’, ‘hill’ and ‘mountain zone’ according to the Federal Office for Agriculture (FOAG, 1999). Swiss regions included the ‘Lake Geneva region’, ‘Espace Mittelland’, ‘Northwestern Switzerland’, ‘Zurich’, ‘Eastern Switzerland’, ‘Central Switzerland’ and ‘Tessin’. Each agricultural enterprise belongs to one main farm type of plant production, livestock farming or combined farming, namely, ‘specialist field crops’, ‘specialist horticulture’, ‘specialist permanent crops’, ‘specialist ruminant livestock’, ‘specialist granivore’, ‘mixed cropping’, ‘mixed livestock’ and ‘mixed crops-livestock’. Furthermore, the barn systems ‘loose housing’ and ‘tie stall’ were included in the analyses as well as ‘both’ if both systems were in use on the farm.

### Statistical analysis

In the first part, frequencies of digital technology adoption were calculated for all livestock-related agricultural enterprises. In the second part, farm and farmers’ characteristics associated with digital technology adoption in ruminant farming were assessed using regression analyses to better understand the adoption process. We focused these analyses on ruminants because all ruminant farmers were asked the same questions and used similar husbandry systems in practice. The classification was based on the first question of the use of ESMDs because ECs and EDPOs require their use.

Based on the results from the first part, the digital technologies were divided into implemented ones that have been already proven in practice and new ones that make farmers pioneers in their use. Thus, three categories were created: The category of implemented technologies includes all technologies used by at least 10% of the farmers surveyed. The category of new technologies includes all technologies used by less than 10%. Because multiple answers were possible, individual farmers can occur in both groups. The third group comprises the non-adopters.

Two binary regression analyses were done to evaluate correlations between farm and farmers’ characteristics and the adoption of implemented and new digital technologies, each compared with the group of non-adopters. For both cases, the dependent variable was the adoption decision (0/1) and the independent variables included the farm and farmers’ characteristics. Estimated marginal changes (dF/dx) in the regression results indicate the change in the probability of adoption when the respective independent variable (clustered at the enterprise level) changes by one unit while keeping all other variables at their averages. The livestock units and age variables are presented in standardised form, that is, expressed in standard deviation differences from the overall sample mean. This presentation allows a meaningful interpretation because the variables contain comparatively large numeric values, so that single-unit changes represent only incrementally small changes compared with the overall spread of the distribution. Results were analysed with the statistical software R Version 3.5.3 (R Core Team, 2013) using the package ‘mfx’ (Fernihough, 2019).

## Results

### Description of respondents

First, the farm and farmers’ characteristics were described for all respondents and for ruminant farming only (Table 1). The farmers were on average 48 years old and predominantly male. All respondents had an average agricultural area of 27 ha and on average 62 livestock units per farm but with high deviations from the mean values. The majority of all respondents managed the farm conventionally and full-time. About half of the farms were located in the valley, followed by mountains and hills. The characteristics of farmers with ruminants differed only slightly from those of all farmers: most of the ruminant farms were located in the mountains, followed by valley and hills. Whereas most of the ruminant farmers (561) kept their animals in loose housing systems, 157 kept them in tie stall barns and 27 had both husbandry systems.

**Table 1:** Farm and farmers’ characteristics of non-respondents and all livestock respondents and of respondents to ruminant farming. Mean values ± SD are shown for numeric variables and total numbers are shown for categorical variables

Variable	Non-respondents (livestock)	All respondents (livestock)	Respondents to ruminant farming
Number (*n*)	1222	1497	832
Age (mean ± SE)	48 ± 10	48 ± 9	48
Total agricultural area (ha) (mean ± SD)	26 ± 33	27 ± 20	24 ± 20
Livestock units total (mean ± SD)	60 ± 57	62 ± 56	40 ± 39
Gender			
Male (0)	1046	1455	798
Female (1)	176	42	34
Production system			
Conventional (0)	1061	1291	660
Organic (1)	161	206	172
Working time			
Part-time (0)	95	112	89
Full-time (1)	1127	1385	743
Zone			
Valley	542	725	272
Hill	189	249	132
Mountain	491	523	428
Region			
Lake Geneva region	108	134	86
Espace Mittelland	373	461	260
Northwestern Switzerland	66	141	62
Zurich	63	68	38
Eastern Switzerland	325	374	214
Central Switzerland	256	273	127
Tessin	31	46	45
Main farm types			
Specialist field crops	15	22	7
Specialist horticulture	7	8	4
Specialist permanent crops	2	5	4
Specialist ruminant livestock	703	741	680
Specialist granivores	270	349	7
Mixed cropping	12	19	10
Mixed livestock	143	226	53
Mixed crops-livestock	70	127	67
Enterprise			
Dairy cattle	160	253	253
Dairy goats	129	136	136
Suckler cows	78	112	112
Beef cattle	259	210	210
Meat sheep	115	121	121
Breeding pigs	140	158	-
Fattening pigs	113	124	-
Laying hens	106	150	-
Broilers	122	233	-
Husbandry system*			
Loose housing	-	-	561
Tie stall	-	-	157
Both	-	-	27

*Information from questionnaires. SE = standard error.

### Frequencies of digital technology adoption

*Ruminants.* Table 2 shows the three questions concerning the adoption of ESMDs, ECs and EDPOs for all ruminant farms. The adoption of digital technologies varied widely depending on the animal species and the type of technology. Compared with farmers in all other ruminant enterprises, farmers with dairy cattle used digital technologies the most, which is illustrated by the answer option ‘none’ being ticked by 32%, 66% and 67% for ESMDs, ECs and EDPOs, respectively, which is considerably less than in the other enterprises.

**Table 2:** Frequencies (%) of adoption of electronic sensors and measuring devices, electronic controls and data-processing options in Swiss ruminant farming

1. Which electronic sensors and measuring devices do you use?						
	Dairy cattle	Dairy goats	Suckler cows	Beef cattle	Meat-sheep	Percentage total
	(*n* = 247)	(*n* = 133)	(*n* = 111)	(*n* = 195)	(*n* = 119)	
None	32	69	84	71	72	60.9
Others	2	3	4	3	3	2.9
Pasture growing measurement	0	0	0	0	na	0
Roughage intake	1	1	0	2	na	1
Animal tracking systems	1	0	1	1	2	1
Rumination sensors	4	0	0	1	na	2
Activity sensors	6	0	0	2	1	3
Electronic ear tags	2	2	5	1	13	4
Electronic weighing system	6	1	5	9	3	5
Camera monitoring	11	1	7	8	10	8
Milk conductivity sensor	12	0	na	na	na	8
Concentrate feed intake	24	2	0	8	3	10
Milk temperature sensor	16	8	na	na	na	13
Transponder collar	26	0	2	14	na	14
Milk flow sensor	26	0	na	na	na	17
Digital milk meter	45	9	na	na	na	32

2. Which electronic controls do you use?						
	Dairy cattle	Dairy goats	Suckler cows	Beef cattle	Meat-sheep	Percentage total
	(*n* = 237)	(*n* = 131)	(*n* = 107)	(*n* = 194)	(*n* = 118)	
None	66	92	94	77	98	82
Others	2	2	2	2	1	2
Automatic feeding system	3	2	2	3	na	3
Selection gates	5	0	1	3	na	3
Automatic calf feeder	12	6	na	13	na	11
Concentrate feeding station	27	2	2	12	1	12

3. Which electronic data-processing options do you use?						
	Dairy cattle	Dairy goats	Suckler cows	Beef cattle	Meat-sheep	Percentage total
	(*n* = 237)	(*n* = 128)	(*n* = 106)	(*n* = 190)	(*n* = 118)	
None	67	88	85	83	82	79
Others	3	0	2	3	0	2
Body condition scoring with camera system	0	1	0	1	0	0
Pasture management	1	1	2	1	1	1
Disease detection	4	1	1	3	0	2
Feed ration planning	6	2	4	6	2	4
Oestrous detection	10	0	1	na	na	5
Data transfer into herd management systems	19	10	10	11	16	14
Concentrate feed allocation depending on milk yield	21	2	na	na	na	15

Na = not applicable.

Dairy cattle farmers most commonly applied easy-to-use digital technologies related to the milking process. For example, digital milk meter was the most frequently used technology, with 45% of the total. Likewise, transponder collar, milk flow sensor and concentrate feed intake were ticked by more than 20% of the dairy cattle farmers.

Digital milk meters were also the most frequently used sensors for dairy goats even though the percentage was considerably lower with 9%. For suckler cows, cameras, electronic ear tags and electronic weighing systems were used the most with frequencies of 5% to 7%. Farmers raising beef cattle ticked transponder collar the most with 14%, followed by electronic weighing systems with 9%. For meat-sheep, electronic ear tags were ticked the most with 13%, followed by camera monitoring with 10%. Regarding the use of ECs and EDPOs, most ruminant farmers ticked ‘none’, but 11% and 12% stated using ECs for the automatic calf feeder and the concentrate feeding station, respectively. Of all the possible answers regarding EDPO use, data transfer into herd management systems was ticked the most with 10% to 19% for all ruminant species.

*Robotics.* Six percent of the farmers surveyed in the dairy cattle sector stated that they had a milking robot and another 6% had a manure removal robot. A robot for automated feed pushing was used by 2% of dairy cattle and beef cattle farmers (Table 3). None of the surveyed suckler cow farmers indicated having an automated feed pusher or manure removal robot.

**Table 3:** Frequencies (%) of adoption of robots in Swiss ruminant farming

	Dairy cattle	Beef cattle
Milking robot	6 (*n* = 239)	na
Automated feed pusher	2 (*n* = 243)	2 (*n* = 199)
Manure removal robot^1^	6 (*n* = 115)	1 (*n* = 138)

1 Only in loose housing not in tie stall barns.

*Pigs and poultry.* The use of electronic ear tags differed strongly between breeding pigs and fattening pigs with 33% and 4%, respectively (Table 4). In poultry farming, differences in smartphone adoption for barn monitoring between the two enterprises laying hens (41%) and broilers (47%) were small (Table 5).

**Table 4:** Frequencies (%) of adoption of electronic ear tags in Swiss pig farming

	Breeding pigs (*n* = 154)	Fattening pigs (*n* = 120)
None	57	94
Others	12	2
Electronic ear tags	33	4

**Table 5:** Frequencies (%) of adoption of barn monitoring in Swiss poultry farming

	Laying hens (*n* = 139)	Broilers (*n* = 231)
Others	19	9
Alarm horn	44	57
Smartphone	41	47
Emergence landline	35	27

### Regression analyses

Farm and farmers’ characteristics associated with digital technology adoption in ruminant farming were assessed using regression analyses. The corresponding marginal effects of the binary logistic regressions are shown in Table 6. The effects for the implemented and new technologies slightly differed. The analyses showed that the type of production (organic or conventional), the working time (full- or part-time business) and the agricultural area were not related to the adoption of digital technologies in ruminant farming in Switzerland. However, farmers with larger numbers of livestock units were more likely to adopt both types of technologies than farmers keeping fewer livestock. On the other hand, age was negatively and significantly correlated to the adoption of new digital technologies: farmers were less likely to adopt this type of technology with increasing age. Furthermore, the results indicated that female farmers were less likely to adopt any type of digital technology compared with male farmers. The zone, the main farm type, the region, the enterprise and the barn system mattered for adoption. More specifically, compared with the base category valley, a small negative effect on the adoption of implemented digital technologies could be found for hill and mountain zones and a strong negative effect on the adoption of new technologies for the mountain zone. Furthermore, compared with the base category dairy cattle, all other ruminant enterprises were less likely to adopt both types of technologies except for the adoption of new technologies for meat-sheep, for which no significant difference could be found. Farmers with animals in tie stall barns and farmers who had a combination of loose housing and tie stall systems were less likely to have implemented technologies compared with the base category loose housing. For the adoption of new technologies, this effect could only be found for tie stall systems.

**Table 6:** Results of the binary logistic regressions on digital technology adoption in ruminant farming. Basic categories in parentheses

	Implemented technologies		New technologies	
Variable	Marginal effect	Standard error	Marginal effect	Standard error
Age	−0.01	0	−0.03**	0.01
Total agricultural area (ha)	0	0	0	0
Livestock units	0.05**	0.02	0.03*	0.01
Gender (male)				
Female	−0.08***	0	−0.08***	−0.02
Production system (conventional)				
Organic	−0.02	−0.03	−0.03	−0.04
Working time (part-time)				
Full-time	0.03	−0.02	0.02	−0.01
Zone (valley)				
Hill	−0.03*	−0.01	−0.04	−0.03
Mountain zone	−0.07*	−0.03	−0.09***	−0.01
Main farm type (specialist ruminant livestock)				
Specialist field crops	0.43**	−0.15	−0.10***	−0.01
Specialist horticulture	−0.08***	0	−0.10***	−0.01
Specialist permanent crops	−0.08***	0	−0.10***	−0.01
Specialist granivore	−0.08***	0	0.03	−0.31
Mixed cropping	−0.09***	0	0.12	−0.16
Mixed livestock	−0.05	−0.03	−0.02	0.06
Mixed crops-livestock	0	−0.02	−0.03	−0.04
Region (Espace Mittelland)				
Lake Geneva region	−0.06*	−0.02	−0.02	−0.01
Northwestern Switzerland	−0.04***	−0.01	−0.06***	−0.02
Zurich	−0.03	−0.03	−0.02	−0.03
Eastern Switzerland	0	−0.01	−0.02	−0.02
Central Switzerland	−0.01	−0.01	−0.06**	−0.02
Tessin	−0.05***	−0.01	−0.06	−0.04
Enterprise (dairy cattle)				
Dairy goats	−0.10***	−0.01	−0.11***	−0.01
Suckler cows	−0.16***	−0.01	−0.06***	−0.01
Beef cattle	−0.12***	−0.01	−0.06***	−0.01
Meat sheep	−0.17***	−0.01	0.01	−0.01
Husbandry system (loose housing)				
Tie stall	−0.10***	−0.01	−0.10***	−0.01
Both	−0.05***	−0.01	0.05	−0.03

Asterisks indicate levels of significance: *P ≤ 0.10; **P ≤ 0.05; ***P ≤ 0.01.

## Discussion

### Frequencies

The differences in adoption pattern between the animal species show that there are areas and production branches in which the use of digital technologies is already commercially implemented. This is mainly the case in the dairy sector. Compared with other livestock sectors, the dairy cattle sector has by far more digital technologies available (Stachowicz and Umstätter, 2020). The milking process is time-consuming and related to a high physical workload, so that the expected advantage of using digital technologies quickly becomes apparent. User-friendly technologies that are integrated, for example, in the milking parlour have higher adoption rates in practice than technologies that collect additional data on the animal or in the barn, for example, for disease detection and that may be bought separately. An exception is the EDPO data transfer into herd management systems, which was ticked by more than 10% of the farmers in each of the enterprises. However, the usage has a direct benefit because many animal-related parameters have to be recorded in general for quality assurance and documentation purposes and are therefore essential for economically viable production. It can be therefore concluded that political incentives can also lead to increased adoption.

Our results confirm the results from other countries for which the use of digital technologies in dairy production has been investigated. A study from New Zealand showed that technologies related to the milking process itself are used more than information collection technologies for example, for disease detection or heat detection (Edwards *et al.*, 2015). Gargiulo *et al.* (2018) evaluated different adoption patterns according to herd sizes among Australian farmers and found that larger farms adopt more precision dairy technologies than smaller ones. In our study, the number of livestock units was also positively correlated to the adoption of digital technologies.

However, our results also show that there are still agricultural enterprises that are managed almost without or with sporadic use of digital technologies. This is especially the case for agricultural enterprises that have a low production value *per se* or where the workload per livestock unit is comparatively low. But even in the dairy sector a considerable share of farmers did not use digital technologies at all. With regard to the high workload for milking, this is a surprising result for a country where dairy farming is very widespread. On the other hand, it is also possible that farmers stated using none of the surveyed technologies but that certain technologies are automatically integrated, for example, into the milking parlour, so that it is not always an active decision to have them and use them.

Barkema *et al.* (2015) investigated the worldwide commercial implementation of milking robots in a comparative study. Their results showed that the use of milking robots varies between 5% in Canada and over 20% in Sweden and Denmark. Almost 6% of the surveyed farmers used a milking robot in our study, thus Switzerland is in the lower international range here. Nevertheless, milking robots are not stand-alone units because they contain a large number of sensors and measuring systems that automatically record and connect data, even if the farmer may not use all available information (Ordolff, 2001). However, the share of farmers using robots on their farms is still very small and mainly limited to dairy farming.

None of the participating farmers in our study indicated using pasture growth measurements, and only 1% stated using data-processing options for pasture management. Also, Gargiulo *et al.* (2018) found low adoption rates for automated pasture measurement in Australia, possibly because it is very time-consuming and difficult to apply.

An international survey regarding the use of PLF technologies in dairying showed that mastitis, nutrition and reproduction were high-priority research topics, whereas goat farming and grassland management ranked as lower priority (Palczynski, 2016).

However, worldwide comparison or ranking of adoption rates for digital technologies is difficult because there is no uniform survey method and almost no representative study. For example, several studies used voluntary online surveys with the selection bias that participants may be relatively technically inclined farmers who use computers and the Internet in general (Gargiulo *et al.*, 2018). Furthermore, the sampling procedure should be representative and cover as far as possible all size classes of farms in order not to overestimate or underestimate the adoption rate. As an example, in a multidisciplinary study by Gargiulo *et al.* (2018), an online questionnaire was distributed using a snowball method among industry contacts and their network. Although 301 questionnaires were received, there were no selection criteria for the survey sample. In our study, we considered almost all Swiss farms for random sampling and stratified the sample for each farm type to ensure that farms of different size classes were addressed. This approach makes our study more significant and representative than most available studies.

Overall, our findings show that production-intensive livestock farming enterprises such as dairy cattle, breeding pigs or poultry often use digital technologies, even if it is not possible to deduce the entire adoption from example technologies for pigs and poultry. However, although only example technologies were surveyed for these two enterprises, trends can still be identified. Considerably more farmers used electronic ear tags for breeding pigs than for fattening pigs. This difference could be due to the different production systems. Breeding pigs are very labour intensive and require a higher level of management, whereas pig fattening involves fewer work processes.

### Farm and farmers’ characteristics

In the present study, an increase in age was associated with a decrease in the likelihood to adopt new technologies, whereas no correlation could be found for technologies already implemented. The number of livestock units as proxy for farm size was positively correlated with both types of technologies even though the effect was stronger for implemented than for new technologies. The agricultural area did not matter for adoption. These findings confirm the inconsistent results from the literature for age and farm size. For example, age and farm size were not associated to the adoption of electronic identification tools for sheep, whereas the likelihood of adoption of nutrient abatement technologies increased with increasing farm sizes and decreased for older farmers (Lima *et al.*, 2018; Konrad *et al.*, 2019). Furthermore, a recent study investigated the adoption of digital technologies among crop, dairy and livestock producers in the USA with the results that size (expressed as hectares and numbers of animals) was positively correlated with Internet access and level of usage and gender (women), farm income and education level (Drewry *et al.*, 2019). In our study, however, female farmers were less likely than male farmers to adopt digital technologies, but the sample included only very few female farmers. Interestingly, our results further showed that farmers using tie stall barns adopted less technology, both implemented and new, compared to farmers using loose housing systems, likely because many technologies do not bring an added value in tie stall barns, where cows cannot express their behavior freely.

The finding that the zone correlated with technology adoption was to be expected and confirms the results of a recent study on the adoption of precision agricultural technologies on Swiss crop farms (Groher *et al*., 2020). Mountain farms in particular often generate less income (FSO, 2019b) and have to cope with difficult production conditions, which may explain the strong negative correlation on new technology adoption. However, small and inexpensive technologies can also support these farms. For example, activity sensors, electronic identification tools or animal tracking can be used to remotely monitor animal behaviour or location. Moreover, precise pasture management could help to use existing resources more efficiently.

Apart from the many opportunities that the use of digital technologies offers, some studies have explored the barriers in the adoption of digital technologies in agriculture (Wathes *et al.*, 2008; Drewry *et al.*, 2019). For instance, a major challenge is the interpretation of the recorded data because the time-varying and individual behaviour of each animal makes an interpretation difficult (Palczynski, 2016). An additional barrier in the adoption of technologies can be the insufficient robustness of sensors (Wathes *et al.*, 2008). Additionally, systems of different manufacturers may be incompatible and a combination of data received from different sensors must be transformed into usable information (Van Hertem *et al.*, 2017). Certainly, the financial advantage is one of the major determinants in the adoption decision (Reichardt and Jürgens, 2009; Pathak *et al.*, 2019). The farmers’ view seems to be that the use of modern technologies and smart farming is very expensive and only profitable for larger farms, maybe due to the perception of high costs and the complexity. However, there are other technologies that are inexpensive, easy to use and do not entail enormous costs (Schrijver *et al.*, 2016). Interestingly, Lima *et al.* (2018) found that users of digital technologies are more likely to see the technologies as useful and practical than non-adopters showing that farmers’ perceptions and beliefs are also important determinants in technology adoption.

### Limitations and benefits

The main focus of this study was to assess the state of automation and mechanisation in Swiss agriculture. Therefore, questions related to digitalisation in agriculture were only one of many parts of the survey with limited scope. The selection of technologies were based on a literature research, always with regard to technologies that were known to be relevant for Switzerland. Although we thoroughly chose these technologies based on these criteria, it is of course possible that some technologies were missed on the list. Furthermore, personal motives to investigate the farmers’ perceptions and possible barriers to adoption of technology were not surveyed and are therefore a possible subject of future research, to further understand the adoption process.

The presented results are mainly in line with the existing literature and low adoption rates are as expected, which we now evidenced by research data. Our article extends the adoption literature by deriving knowledge from survey data combining a representative random sampling procedure with a considerably large response rate, which provides us a representative picture of the overall farming population in Switzerland. Technology adoption, especially of digital technologies, is evolving over time. Therefore, it is beneficial to study the overall adoption rate in different countries or regions to get an up-to-date view on current developments that can be used to derive knowledge on determinants for technology uptake.

## Conclusion

The adoption of digital technologies in Swiss livestock farming varies strongly between different agricultural enterprises and is most common on large specialist ruminant livestock farms. In general, easy-to-use sensors and measuring devices, for example, integrated in the milking parlour are more widespread than data-processing technologies. The husbandry system also determines the use of digital technologies, with the result that farmers with tie stall barns are less likely to use digital technologies than farmers with loose housing systems. Studies of farmers’ personal determinants of adoption and prospects of implementation can help identify further barriers to the adoption of digital technologies.

## Software and data repository resources

None of the data were deposited in an official repository.
